# Observations on Two Forms of Dysmenorrhœa

**Published:** 1847-04

**Authors:** Henry Oldham

**Affiliations:** Physician-Accoucheur and Lecturer on Midwifery at Guy’s Hospital


*Observations on two Forms of Dysmenorrhœa.* By Henry Oldham, Physician-Accoucheur and Lecturer on Midwifery at Guy's Hospital.

Case.—*Painful but copious menstruation—dysmenorrhœal membrane—large and retroverted womb—bleeding—mercury—cure.*—Mrs. G., æt. 31, living at Bow, was pregnant soon after marriage, and bore a living child, which is now nine years old. Since this time she has had several early abortions at the fifth or sixth week: and a year ago, when pregnant, she was much frightened at an accident to her husband, and again miscarried. Both before and since this time she has suffered a great deal from pains in the back and hips, for which she has been under medical treatment.

She first consulted me on Nov. 20th, 1844, when she was evidently suffering severely. Her countenance was distressed; she walked with pain, and appeared to be much out of health. Her symptoms were referred almost entirely to the uterus. She complained of habitual pain, which she called painful pressure, at the sacrum, running down to the anus ; constant pain in the right inguinal canal ; occasional dysuria, which, with the other pelvic symptoms, was much increased by walking or exertion of any kind. When she sat down their was pain produced within the pelvis. The bowels were relieved without pain. A copious thick discharge, sometimes white and at others yellowish, had troubled her for years. Sexual coitus had caused much suffering, and, in fact, had been abandoned. She menstruates regularly, but with severe pain ; sometimes copiously, so that several clots escape, and sometimes shreds of membrane have been noticed : she feels better and lighter after the periods.

On examination, the vagina was found loose and hotter than usual, and freely bathed with discharge. The posterior wall bulged a little beyond the ostium vaginæ. The uterus was found within two inches of the orifice of the vagina : the vaginal portion was tumid, soft, slightly painful to the touch ; and the os, which was patent and uneven, gave that feeling of raised soft granulations which characterizes the most common form of ulceration of this part. This portion of the womb was directed towards the under surface of the urethra. The body of the womb was readily felt backwards towards the hollow of the sacrum. It was much swollen, very tender when touched, and was evidently retroverted. On directing the finger to the front part of the cervix, and pressing the part downwards and backwards, the body of the womb was readily redressed, and she felt at once a relief from the painful sense of pressure in the sacrum and hips. When the finger was removed the womb fell heavily back again towards the sacrum. By speculum the cervix showed a surface of vividly injected granulations, and a large plug of mucus was pressed out from its cavity.

The treatment in this case consisted in scarifying the cervix, and thus bleeding the womb ; taking from one to two ounces of blood at a time, which was repeated on several occasions. She was kept at rest ; the bowels were relieved by saline aperients, and she used the following injection :—Dec. Papav. vj. ; Ext. Conii. ℈j. ; Liq. Plumb. diac. ʒij. Speedy relief followed the use of these means, and I did not see her for some time.

In July, 1845, she brought me a portion of membrane, which had been passed at the last period, after severe pains like labour pains ; and on examining it I found it was a good specimen of dysmenorrhœal membrane. She told me at the same time that she was convinced that in several of her supposed early abortions she had passed the same product. On two or three occasions after this, she brought me portions of membrane which were cast off after severe suffering at the menstrual time, which were now often deferred for a few days or a week beyond their proper period. On the 10th of February, 1846, after a period of intense pain, she again passed some detached portions of membrane. She had bad difficulty in emptying the bladder, which was relieved by her passing water in the upright position. The uterus was found retroverted, and the body was swollen into a large globular hardened mass, painful to the touch. Any exertion produced great pain in the sacrum and hips, which was again at once relieved by redressing the womb during a vaginal examination.

I now determined to leech the uterus once a week, to keep her strictly at rest, and to put her under a course of mercury. The effect of this was to reduce the womb, and to give her great relief. She passed through the two following periods without much suffering, and no membrane was cast off. On the 30th of May she brought me two small portions of membrane, which had caused her some severe pain when passed. The seat of pain was in or about the right inguinal canal, and she said that she could touch the spot from whence she fancied the membrane was torn away. The remainder of the period was passed without pain. The uterus was now very much reduced in size, and although more vertical than it should be, it was not inclined backwards. She is able to walk and follow her usual pursuits without pain. A blister was applied to the sacrum, and a little mercurial ointment was rubbed at night over the painful part. Since this period she has menstruated with comparative comfort, and the womb, although perhaps rather large, may be said to be fairly healthy and well placed.

This is a case which in its general features is frequently met with in practice, and it may be very well taken to illustrate the pathology, symptoms, and treatment, of the membraneous form of dysmenorrhœa. It will be seen that painful menstrual periods, with a copious rather than a scanty flow, the unfolding and casting off of portions of membrane, then a large heavy indurated womb, the weight of which destroys its balance and displaces it backwards, are the principal signs to be noticed. Partial prolapse too, congestion and ulceration of the cervix, with sterility, may also be remarked. It is not my design in this communication to treat at full length the subject of membraneous dysmenorrhœa, but a few remarks on the sequence of morbid actions in this class of cases, with the treatment which is indicated, will, I hope, be thought useful.

The membrane which is thrown off from the womb in this disease varies in its appearance; sometimes a clear tubular cast of the uterine cast is expelled, although this, I believe, is comparatively rare. More frequently, small portions the size of the nail, or larger pieces of an irregular shape, are detached, and cause severe suffering in their expulsion. Sometimes the menstrual blood coagulates around a portion of membrane, and comes away as a hard compact clot. Very often the discharge consists of thin thread-like portions, and sometimes half a cupful or more of thick branching tufts, very much like the chorion villi filled with blood, will be thrown out from the womb. This latter variety of discharge has, I believe, been mistaken for the so-called hydatid degeneration of the placenta, which I feel persuaded never takes place unless impregnation has preceded it, and a true chorion formed. How is this membrane produced? It is generally thought to be lymph, but if some good specimens are carefully examined, they will be found to possess the same structural elements as the uterine decidua. Not only do they resemble the decidua in having an attached rough surface and a smooth free one, but what is far more significant of their identity is, that they are full of little holes with epithelial scales, which I cannot doubt are the openings and epithelium of the follicles of the uterine glands. It is very true, as Dr. Montgomery has noticed, that the small cotyledonous sacs are wanting. But this is often the case in the flaps of uterine decidua which are thrown off in abortions, although here and there very perfectly formed specimens of them are to be found. I doubt whether the swollen uterine glands bulge out into sacs until the ovum gets fixed by its exochorion within them, as, in two specimens of extra-uterine fœtation which I have examined, the very exuberant decidua, with its characteristic apertures and epithelium, still remains tubular without expanding into cells. I have for some time entertained the conviction that the membrane cast off in dysmenorrhœa is formed from the enlarged uterine follicles, just in the same way as is the uterine decidua, and that, like it, it is detached from the cavity of the womb. The shred-like masses are caused by a very recent and imperfectly formed membrane, which breaks up and becomes mixed with and stained by the menstrual blood.

The practical bearing of these remarks is, that as the uterine decidua is formed under the influence of an action going on in the ovary, so the membranous dysmenorrhœa is not primarily an affection of the womb, but of the ovary. In healthy menstruation the congestion of the ovary, the engorgement of the womb, the opening of the veins on the surface of the cavity of the womb, and the flux of blood, are all in harmony, the latter being, so to speak, the resolution of the former. But when the ovaries are unduly excited, as, for instance, from the prevalence of one or more of the numerous ways in which sexual feelings may influence them, then the uterine glands sympathetically enlarge, the lining membrane of the womb becomes raised, and the body of the womb swells out. This change in the mucous membrane goes on during the interval between the monthly periods, and when the flow begins the new formation is cast off, and the uterus in the act of detaching and expelling it becomes the seat of very painful contractions. Gendrin, Jörg, and others, have enumerated amongst the changes which are observed in the internal sexual organs during menstruation, that the lining membrane of the womb is covered with fungiform villi which are probably vascular. And this observation would seem to be related to Müller's idea, "that menstruation is the result of a periodical regeneration, a kind of moulting of the female generative organs, attended perhaps with the formation of a new epithelium." I lately examined the uterus of a young female who died menstruating, but the mucous membrane was still clear and smooth. Blood exuded freely when the substance of the womb was pressed, but there was no appearance of that raising of the lining of the membrane of the womb which is erroneously described as from fungiform villi. I very much doubt whether this change, and the consequent periodical moulting of the mucous membrane of the uterus, occurs uniformly during menstruation. It is speedily produced, however, under any morbid excitement of the ovaries, and is then cleared off either in threads, or thin flimsy portions, or in larger and denser patches, according to the degree of developement at which it has arrived.

But there is a sequel of the membraneous form of dysmenorrhœa which merits more attention, namely, the tendency of the womb to become permanently bulky and hard, and as the result of this to become retroverted. I can bear testimony to the truth of an observation of Dr. Rigby, that retroversion is one of the most common affections of the unimpregnated womb, and I would add, that one amongst several causes which produces it is the continuance of this membranous dysmenorrhœa. It will be noticed in the case I have related, and it is a mark of distinction between this and obstructed dysmenorrhœa, that at first a copious menstrual flow took place—menorrhagia, in short. This symptom, while it shows the way in which two different functional disorders of the womb are associated together or run into one another, is, I believe, a salutary effort to relieve the morbid congestion of the uterus. Like hæmorrhage from other organs when diseased, it is really conservative, a useful topical bleeding. But after a time the uterus does not recover itself, it becomes heavier and larger, and it appears that the posterior wall swells out more than the front wall, and then the womb loses its natural inclination forward; it first becomes vertical, then inclined backward, and at last retroverted. This change occurs slowly, sometimes taking many months to accomplish. The texture of the womb becomes altered. In a recent congestion the posterior wall is felt soft, compressible, and painful to the touch, but after repeated engorgements the tissue becomes harder, more solid, very much like a fibrous growth. A further change too I have noticed, which is, that occasionally when the womb is thus displaced, it excites inflammation in the neighboring peritoneum, false membranes are formed which fix the womb, and an irreducible retroversion is the result.

I have laid some stress on the swelling of the posterior wall, because it appears to me to be more sensibly affected by congestion than the anterior wall. The natural convexity of this part becomes still more prominent, and, when examined by the finger, it often feels so round and solid, and swells out so abruptly from the cervix, that I am quite sure that it is often mistaken for a fibrous tumour. This swelling of the posterior wall forms a good practical distinction between a womb enlarged by congestion and a womb distended by an early pregnancy. I have been in the habit of depending very much on the even enlargement of the anterior wall of the womb, which is quite appreciable to the finger, as a good diagnostic mark of an early pregnancy. The natural flatness of the anterior wall is quickly effaced by pregnancy; so that, so early as a month or five weeks, this physical change may be detected.

The symptoms which attend this form of retroversion are not, generally speaking, so severe or so well marked as might be expected, or, indeed, as they are described. It is rare for the displaced womb mechanically to interfere with the bladder or the rectum, and as rare for it to press upon and obstruct the veins. Sometimes, however, if a person has been standing for any length of time, especially during menstruation, dysuria ensues, which is relieved by lying down for some hours. The rectum, too, in these cases, generally escapes pressure; and it is surprising, even when the womb is very solid and bulky, appearing to the finger to fill the cavity of the sacrum, how rarely mechanical constipation ensues. In these cases, as in pregnancy, there seems to be some mechanism, which, I think, notwithstanding what has been said about it, is yet unexplained, by which the uterus is directed towards the right side of the pelvis, removing it from the rectum. There are, however, a class of cases where engorgement of the womb is associated with hæmorrhoids and bleeding from the rectum, which ought not to be confounded with the present cases; they occur in women whose digestive organs have been long disordered,—who indulge freely in eating and drinking, and take but little exercise,—whose bowels are habitually constipated, and the colon loaded. The urine in these cases is often charged with lithic acid, and, as a consequence of the portal congestion, the hæmorrhoidæl veins swell, and eventually the uterus becomes engorged. Painful and oftentimes copious menstruation occurs in this form of complaint, as Dr. Rigby has so well shown; but the pathology of the two classes of cases is essentially different. In the one, the depraved state of the digestive organs induces engorgement of the womb; in the other, the affection is at first confined to the internal sexual organs, and any disorder of the general health is slowly induced.

The principal symptoms which I have noticed as the result of the large and retroverted womb have been, an habitual weight in the lower part of the abdomen, and a painful sense of pressure about the sacrum; pain of a dragging kind referred very distinctly to the inguinal canals, but very often only to one of them, with pain in the corresponding hip. There is pain, too, in sitting down, and a feeling as though some body were pressed upwards. If the cervix gets bulky and full, a thick mucous discharge comes on, which frequently is discharged in lumps.

It has been advised in these cases to redress the womb by means of the uterine sound; but I think this expedient is very rarely required. If the forefinger is placed against the anterior part of the cervix, and this part pressed backward, the womb, although large and heavy, may readily be raised and directed forward, and, in the act of reducing it, a sense of its weight and the extent of its displacement is conveyed to the finger. No permanent good, however, is obtained by the operation, as the womb quickly falls back again, and the same symptoms of pressure, which for the moment were relieved, again come on. I have known a great deal of pain caused by using the sound in the way described; and, if the womb is held by false membranes, to attempt to overcome the resistance by the sound would be not only painful but dangerous.

In the treatment of membranous dysmenorrhœa, with a large, hard, retroverted womb, I have found the plan which was adopted in the case described the most effective. It consists in keeping the patient as much at rest as possible, in regulating the diet so as generally to avoid stimulants, but not to lower the strength, and to give small doses of mercury, so as slightly to affect the gums: two grains of blue-pill, with three of Ext. Conii, night and morning, or five-grain doses of Plummer's pill night and morning, answer the purpose required. On the first appearance of the gums or palate being tender, I generally give the Liq. Hydrarg. Bichlorid. ʒj. in sarsaparilla or bark, which, while it keeps up the action of the mercury, is really a tonic, and improves the general health. If the patient does not bear mercury well, it is well to commence and continue with this last preparation, and a small quantity of blue ointment, with Ext. of Belladonna, may be rubbed at night over the inguinal region. Leeches should be applied to the upper and back part of the vagina once a week. Three or four will in general be sufficient, and I can safely say that they are easily applied, and offer the most effective means that I know of for reducing the womb. Cupping on the loins, or leeching the inguinal regions, are, in my experience, far less useful for the purpose. In the case which I have related the cervix was scarified several times because of the vascular granulations which covered it; but unless a surface of this kind is present, which bleeds freely on being lightly cut, I do not think it a good plan of local depletion. When the size of the womb is reduced by these means, an occasional blister on the sacrum will be found very useful. I have generally found patients obtain great relief by their application. The reduction of the womb may be assisted by warm hip-baths, or the injection of warm poppy-water into the vagina. By following out this plan of treatment, and attending to the general health, I have often succeeded in reducing these large, solid, hypertrophied wombs, with the dysmenorrhœa which accompanied them, and the conviction has forced itself upon me, that the cases of fibrous tumour which have been supposed to be cured have really been cases of this kind. When the influence of mercury is obtained, small doses of Iodide of Potassium, or, what I have found more useful, small doses of the Liq. Potass. Arsen., may be given with advantage. The latter medicine should be administered in the way recommended by Mr. Hunt—on a full stomach, and in decreasing doses.

Sometimes the womb has so long been displaced, and is so very hard, that it seems to resist any attempts at cure. I have now a case of this kind under care in a woman æt. 48, who has ceased to menstruate for a year and a half. In this, as in similar cases, the metallic stem support described by Dr. Simpson effectually retains the womb *in situ*, but I have not found this instrument desirable in other cases.

The object generally is not to support a large womb, but to lessen its size, to stop the ovarian excitement which is causing it, and to secure its spontaneous reduction. In these cases much comfort is obtained by an elastic abdominal belt, with a perinæal support.

The views which have been propounded on this subject may be expressed in the following conclusions:—

1. There is one form of menstruation rendered extremely painful from the production and casting off of a membrane from the cavity of the womb.

2. That this membrane is not a product of inflammation, or a thick mass of epithelium, but it is formed from the uterine glands just as the decidua is, and is detached and expelled in the same way.

3. That the morbid action does not begin at the uterus, but in the ovary; and the sequence of effects is first ovarian congestion, calling forth a sympathetic growth of the uterine glands, forming a false decidua and uterine engorgement.

4. That this uterine engorgement is oftentimes relieved by a profuse menstrual flux; but if not, the posterior wall of the womb gradually increases in bulk and becomes hard, the balance of the womb is spoiled, and the body falls back, retroverting the womb.

5. That the swelling of the posterior wall, and the falling back of the womb, forms a differential diagnosis between congestion and early pregnancy, the anterior wall enlarging in the latter, and the body of the womb directed forward.

6. That the symptoms of retroverted womb from this cause are not often those of mechanical obstruction to the other pelvic viscera, and they are for the moment relieved by redressing the womb, which may almost always be effected by the finger without the aid of the sound.

7. That the treatment consists in strict attention to the general health, but that the most effectual way of removing the disease with the enlarged womb is by leeching the uterus, and the use of mercury.—*London Med. Gaz.*

